# Supplementary material for: A Changing Home: A Cross-Sectional Study on Environmental Degradation, Resettlement and Psychological Distress in a Western German Coal-Mining Region
Source: Int J Environ Res Public Health. 2022 Jun 10;19(12):7143. doi: 10.3390/ijerph19127143 (PMC9223024; doi:10.3390/ijerph19127143)
Supplement: Supplementary file 1 [file ijerph-19-07143-s001.zip › ijerph-1750472-supplementary.pdf]

Supplementary Material

A Changing Home: a Cross-Sectional Study on Environmental Degradation, Resettlement and Psychological Distress in a Western German Coal-Mining Region

T. Krüger, T. Kraus, A. Kaifie

Supplementary Table S1. Intercorrelations Patient Health Questionnaire (PHQ)

	new villages	old villages	pit-edge villages	*p*-Value
		r (*n*)		
somatization and general anxiety	0.79 (167)	0.73 (108)	0.72 (308)	< 0.0001
somatization and depression	0.83 (168)	0.82 (108)	0.74 (309)	< 0.0001
depression and general anxiety	0.92 (167)	0.84 (108)	0.86 (308)	< 0.0001

r = Pearson correlation coefficient

Supplementary Table S2. Correlations between Period since completed Resettlement and Solastalgia / PHQ

	new villages	*p*-Value
	r (*n*)	
solastalgia and period since completed resettlement	0.07	0.3264
somatization and period since completed resettlement	- 0.01	0.8551
depression and period since completed resettlement	0.02	0.8138
general anxiety and period since completed resettlement	- 0.02	0.8060

r = Pearson correlation coefficient

Supplementary Table S3. Sociodemographics, Solastalgia and PHQ Scores and Correlations (w/ exact values)

	new villages	old villages	pit edge villages	Test Value *	*p*-Value
			Sociodemographics		
			mean (SD)		
age	55.7 (15.7)<br>*n* = 173	54.2 (18.1)<br>*n* = 104	53.9 (15.3)<br>*n* = 301	1.93 #	0.3806
			n (%)		
female gender	93 (52.0)<br>*n* = 179	57 (51.4)<br>*n* = 111	177 (55.7)<br>*n* = 318	5.82	0.2127
marriage or partnership	156 (86.7)<br>*n* = 180	75 (67.6)<br>*n* = 111	261 (81.1)<br>*n* = 322	16.08	0.0003
university degree	32 (18.7)<br>*n* = 171	26 (25.0)<br>*n* = 104	69 (22.9)<br>*n* = 301	6.34	0.1754
children living in the village	85 (48.3)<br>*n* = 176	53 (46.9)<br>*n* = 113	130 (40.3)<br>*n* = 323	3.54	0.1701
grandchildren living in the village	27 (15.3)<br>*n* = 176	9 (8.0)<br>*n* = 113	31 (9.6)<br>*n* = 323	5.12	0.0773
former generations living in the region	106 (62.0)<br>*n* = 171	81 (73.6)<br>*n* = 110	188 (60.5)<br>*n* = 311	6.28	0.0434
living on old family property	86 (50.0)<br>*n* = 172	72 (63.7)<br>*n* = 113	131 (41.7)<br>*n* = 314	16.40	0.0003
ownership of residence	158 (89.8)<br>*n* = 176	98 (89.1)<br>*n* = 110	267 (84.8)<br>*n* = 315	3.02	0.2208
spend entire life in the village	72 (40.9)<br>*n* = 176	56 (50.0)<br>*n* = 112	117 (37.1)<br>*n* = 315	5.67	0.0587
			Solastalgia (score)		
			mean (SD)		
	n = 170	*n* = 111	*n* = 312		
solastalgia	21.19 (7.51)	25.59 (5.81)	25.38 (4.97)	40.35 #	< 0.0001
- male	20.09 (7.88)	25.85 (5.73)	24.35 (5.93)	25.34 #	< 0.0001
- female	22.34 (6.94)	25.90 (5.27)	26.32 (3.73)	17.95 #	0.0001
			Patient Health Questionnare (score)		
			mean (SD)		
somatization	6.07 (6.71)	10.28 (7.17)	10.05 (7.04)	43.90 #	< 0.0001
- male	6.01 (6.56)	8.69 (6.98)	8.89 (6.86)	12.18 #	0.0023
- female	6.09 (6.86)	11.98 (6.84)	11.16 (7.03)	35.63 #	< 0.0001
generalized anxiety	4.60 (5.91)	8.92 (6.07)	7.32 (6.64)	52.62 #	< 0.0001

- male	4.20 (5.24)	7.73 (6.23)	6.22 (5.33)	16.80 #	0.0002
- female	4.99 (6.50)	10.19 (5.63)	8.37 (5.70)	37.03 #	< 0.0001
depression	5.02 (6.48)	7.85 (5.86)	7.35 (6.03)	36.39 #	< 0.0001
- male	4.78 (5.98)	7.10 (5.88)	6.50 (6.11)	10.62 #	0.0049
- female	5.22 (6.96)	8.70 (5.68)	8.19 (5.87)	29.23 #	< 0.0001
	Patient Health Questionnare (dichotomized score > 9)				
			n (%)		
somatization > 9	47 (28.0)<br>*n* = 168	58 (52.7)<br>*n* = 110	145 (46.5)<br>*n* = 312	21.24	< 0.0001
- male	21 (26.3)<br>*n* = 80	24 (46.2)<br>*n* = 52	54 (39.7)<br>*n* = 136	6.27	0.0436
- female	25 (29.1)<br>*n* = 86	33 (60.0)<br>*n* = 55	88 (52.4)<br>*n* = 168	16.77	0.0002
generalized anxiety > 9	31 (18.6)<br>*n* = 167	49 (45.4)<br>*n* = 108	96 (31.2)<br>*n* = 308	22.66	< 0.0001
- male	12 (15.2)<br>*n* = 79	17 (33.3)<br>*n* = 51	30 (22.4)<br>*n* = 134	5.88	0.0529
- female	19 (22.1)<br>*n* = 86	31 (57.4)<br>*n* = 54	65 (39.2)<br>*n* = 166	18.02	0.0001
depression > 9	35 (20.8)<br>*n* = 168	37 (34.3)<br>*n* = 108	94 (30.3)<br>*n* = 309	7.13	0.0283
- male	16 (20.0)<br>*n* = 80	17 (33.3)<br>*n* = 51	33 (24.3)<br>*n* = 135	3.01	0.2224
- female	18 (20.9)<br>*n* = 86	19 (35.2)<br>*n* = 54	60 (36.1)<br>*n* = 166	6.42	0.0403
	Patient Health Questionnare and Solastalgia (correlations)				
			r (*n*)		
somatization and solastalgia	0.54 (166)	0.44 (109)	0.42 (310)		< 0.0001
generalized anxiety and solastalgia	0.51 (165)	0.49 (107)	0.38 (306)		< 0.0001
depression and solastalgia	0.53 (166)	0.45 (107)	0.35 (307)		< 0.0001

Respondents from new villages were asked to refer to their village prior to resettlement if necessary; * chi-square or (#) Kruskal–Wallis H test; SD = standard deviation; n.s. = not significant; r = Pearson correlation coefficient

Supplementary Table S4. Experienced or expected Impacts of Resettlement (w/ exact values)

	new villages	old villages	Test Value #	*p*-Value
	n (%)*			
feeling physically exhausted	62 (36.3%) n = 171	64 (59.3%) n = 108	14.14	0.0002
feeling psychologically exhausted	56 (33.0%) n = 170	76 (69.7%) n = 109	36.05	< 0.0001
feeling well informed/advised by authorities	55 (32.2%) n = 171	9 (8.3%) n = 109	21.58	< 0.0001
(expectation of) better general living conditions	92 (53.8%) n = 171	18 (16.7%) n = 108	38.22	< 0.0001
(fear of) lost contact with cherished people	37 (21.6%) n = 171	55 (50.9%) n = 108	25.70	< 0.0001
(fear of) extra financial burden	73 (42.7%) n = 171	76 (71.0%) n = 107	19.85	< 0.0001
(fear of) worse professional situation	12 (7.0%) n = 170	31 (30.4%) n = 102	26.08	< 0.0001
my pets can be kept equally well	58 (73.4%) n = 79	23 (36.5%) n = 63	6.04	0.0140
my livestock can be kept equally well	4 (28.6%) n = 14	1 (3.3%) n = 30	25.44	< 0.0001

* Respondents who strongly agree or agree or (for the last two items: pets/livestock) who indicated yes; # chi-square test

UNIKLINIK RWTHAACHEN
Institut für Arbeits-, Sozial- und Umweltmedizin

Allgemeine Informationen zur Studie

Worum geht es? Im Rahmen meiner Doktorarbeit möchte ich untersuchen, inwiefern sich die Veränderungen von Heimat und Umwelt im Rheinischen Braunkohlerevier auf Gesundheit und Wohlbefinden der betroffenen Menschen auswirken.

Teilnahmevoraussetzungen: Sie sind mindestens 18 Jahre alt und aktuell in einer Ortschaft in der Nähe des Tagebaus Garzweiler II oder Hambach wohnhaft oder waren vor Ihrer Umsiedlung in den vergangenen Jahren dort wohnhaft.

Zeitdauer: Die Bearbeitung dieses Fragebogens wird etwa 15 bis 20 Minuten dauern.

Anonymität: Alle Angaben, die Sie hier machen, werden anonym gespeichert und verarbeitet. Das bedeutet, dass zu keinem Zeitpunkt irgendwelche Angaben auf Sie persönlich zurückgeführt werden können.

Kontakt: Bei Fragen, Problemen oder Ähnlichem können Sie mich gern kontaktieren unter [redacted] oder [redacted].

Mit dem Ausfüllen dieses Fragebogens bestätigen Sie, dass Sie die oben angegebenen Informationen gelesen und verstanden haben und mit der anonymen Verarbeitung Ihrer Daten einverstanden sind.

Wichtige Hinweise zur Bearbeitung der Fragen

Verlassen Sie sich auf Ihre erste Reaktion und beantworten Sie die Fragen spontan.

Es gibt keine richtigen oder falschen Antworten. Bitte antworten Sie so, wie es für Sie am besten zutrifft. Bitte antworten Sie offen und ehrlich.

Bitte lassen Sie keine Frage aus. Sollten Sie einmal nicht die perfekt passende Antwort finden können, so wählen Sie bitte diejenige, die am ehesten auf Sie zutrifft.

Wenn Sie weiterführende Anmerkungen oder Rückmeldungen zum Fragebogen machen möchten, nutzen Sie dafür bitte das Freitextfeld auf der letzten Seite.

UNIKLINIK RWTHAACHEN
Institut für Arbeits-, Sozial-
und Umweltmedizin

Wie alt sind Sie?

…………. Jahre

Welchem Geschlecht ordnen Sie sich zu?

- o männlich
- o weiblich
- o divers
- o Ich möchte keine Angabe machen

Welche Angabe trifft auf ihren Familienstand zu?

- o ledig
- o in einer Partnerschaft
- o verheiratet
- o verwitwet
- o geschieden

Welcher ist Ihr höchster Schulabschluss?

- o Haupt- oder Volksschulabschluss
- o Abschluss der Realschule oder Polytechnischen Hochschule
- o Fachhochschulreife/Abschluss einer Fachoberschule
- o Abitur
- o Schule beendet ohne Abschluss

Welchen höchsten beruflichen Ausbildungs- bzw. Hochschulabschluss haben Sie?

- o Schüler:in
- o noch in beruflicher Ausbildung (Auszubildende:r, Student:in, Berufsvorbereitungsjahr)
- o keinen Berufsabschluss und nicht in beruflicher Ausbildung
- o beruflich-betriebliche Ausbildung (Lehre) abgeschlossen
- o beruflich-schulische Ausbildung (Berufsfachschule, Handelsschule) abgeschlossen
- o Ausbildung an einer Fachschule, Meisterschule, Technikerschule, Berufs- oder Fachakademie abgeschlossen
- o Bachelor an (Fach-)Hochschule abgeschlossen
- o sonstiger Abschluss an einer Fachhochschule oder Ingenieurschule
- o sonstiger Abschluss an einer Universität oder Hochschule
- o anderer Bildungsabschluss

Ich wohne aktuell oder wohnte in den vergangenen Jahren in einer Ortschaft in unmittelbarer Nähe (< 7 km Luftlinie) zum …

- o Tagebau Garzweiler II
- o Tagebau Hambach

Was trifft am ehesten auf Ihre momentane Wohnsituation zu?

- Ich bin vor ……….. Jahr(en) und ………. Monaten umgesiedelt und …
  - ich lebe in einer der Neusiedlungen (z.B. Keyenberg (neu)).
  - ich lebe nicht in einer der Neusiedlungen.
- Ich lebe noch in einer Ortschaft mit Umsiedlerstatus (z.B. Keyenberg) und ...
  - ich habe bereits eine Einigung über meine Umsiedlung mit dem Tagebauunternehmen erzielt.
  - ich habe bisher nur Verhandlungen mit dem Tagebauunternehmen geführt.
  - ich habe bisher keinen Kontakt zum Tagebauunternehmen.
- Ich lebe in einer Ortschaft am Tagebau ohne Umsiedlerstatus, nämlich …
  - Wanlo
  - Kaulhausen / Venrath
  - Holzweiler
  - Morschenich
  - andere: …………………

1. Die folgenden Fragen beziehen sich auf das **Leben in Ihrer ursprünglichen Ortschaft**. Sollten Sie ggf. umgesiedelt sein, beziehen Sie sich bei der Beantwortung bitte auf Ihre vorherige Ortschaft.

Wie lange leb(t)en Sie in Ihrer Ortschaft?

- mein gesamtes Leben
- nicht mein gesamtes Leben, sondern ………… Jahre

Wie viele Generationen Ihrer Familie lebten schon im Rheinischen Revier?

- schon die Generation vor meinen Urgroßeltern
- meine Urgroßeltern
- meine Großeltern
- meine Eltern
- Ich bin in meiner Generation in die Region gezogen

Leb(t)en Sie in einem Haus, einer Wohnung oder auf einem Grundstück, das von früheren Generationen Ihrer Familie bewohnt wurde?

- ja
- nein

Ich wohn(t)e in einem/r…

- Haus / Wohnung im eigenen oder familiären Besitz
- Haus / Wohnung zur Miete

UNIKLINIK
RWTHAACHEN
Institut für Arbeits-, Sozial-
und Umweltmedizin

Haben Sie Kinder? Wenn ja, wo leben diese? (Mehrwachauswahl möglich)

- Ich habe Kinder,
  - die im gleichen Haushalt leben wie ich.
  - die in der gleichen Ortschaft leben wie ich.
  - die in der näheren Umgebung leben (< 10 km).
  - die weiter entfernt leben (> 10 km).
- Ich habe keine Kinder.

Haben Sie Enkelkinder? Wenn ja, wo leben diese? (Mehrwachauswahl möglich)

- Ich habe Enkelkinder,
  - die im gleichen Haushalt leben wie ich.
  - die in der gleichen Ortschaft leben wie ich.
  - die in der näheren Umgebung leben (< 10 km).
  - die weiter entfernt leben (> 10 km).
- Ich habe keine Enkelkinder.

2. Folgend finden Sie **Umgebungsbelastungen durch den Tagebau**, die Sie in Ihrer Ortschaft möglicherweise erlebt haben. Bitte geben Sie an, wie oft Sie diese erlebt haben.

	nie	selten	manchmal	oft	fast immer
**Staub (z.B. Kohlestaub)**					
**Lärm durch Tagebauaktivitäten (z.B. Lastwagen)**					
**Vibration oder Erschütterung durch Tagebauaktivitäten (z.B. Schaufelradbagger)**					
**Lärm durch Umsiedlungen (z.B. Häuserabriss)**					
**Vibration oder Erschütterung durch Umsiedlungen (z.B. Häuserabriss)**					
**Nächtliche Lichtbelästigung durch Tagebauaktivitäten**					
**Erhöhtes Verkehrsaufkommen (z.B. durch Umleitungen)**					

Haben Sie noch andere Umgebungsbelastungen erlebt? Bitte geben Sie diese hier an (optional):

Haben Sie darüber hinaus Bergschäden an Wohnung/Haus oder Grundstück erlebt?

- ja
- nein

3. Folgend finden Sie Aussagen zu den **persönlich empfundenen Auswirkungen des Tagebaus**. Bitte geben Sie an, inwieweit diese Aussagen für Sie zutreffen/nicht zutreffen.

	trifft zu	trifft eher zu	teils-teils	trifft eher nicht zu	trifft nicht zu
**Ich habe Verständnis für den Ausbau des Tagebaus.**					
**Die wirtschaftlichen Vorteile des Tagebaus (z.B. Arbeitsplätze) für die Region sind wichtig.**					
**Ich kann/konnte das Leben wegen der Auswirkungen des Tagebaus nicht so sehr genießen, wie ich es gern möchte.**					
**Meine Ortschaft ist/war durch Meinungsverschiedenheiten über den Tagebau gespalten.**					
**Meine Familie ist/war durch Meinungsverschiedenheiten über den Tagebau gespalten.**					
**Ich bin verärgert über die Zerstörung von historischen Gebäuden und Wahrzeichen durch den Tagebau.**					
**Ich bin verärgert über die Zerstörung von Wohnhäusern durch den Tagebau.**					
**Ich bin verärgert über die Zerstörung von natürlichem Lebensraum für Pflanzen und Tiere durch den Tagebau.**					
**Es stört mich, dass bei der Erweiterung des Tagebaus künftige Generationen nicht mehr mitbedacht werden.**					
**Die Finanzierung von Gemeinschafts-projekten durch das Tagebau-unternehmen ist hilfreich für die Region.**					
**Ich bin besorgt, dass meine Gesundheit durch den Tagebau bedroht sein könnte.**					
**Ich fühle mich machtlos gegenüber den Veränderungen meiner Heimat.**					
**Ich bin zufrieden mit den Bemühungen der Behörden, die Umweltfolgen des Tagebaus zu überwachen.**					
**Die Auswirkungen des Tagebaus in der Region deprimieren mich.**					
**Durch den Tagebau und die damit einhergehenden Veränderungen, wurde mein Gefühl, zu dieser Ortschaft zu gehören, beeinträchtigt.**					

	trifft zu	trifft eher zu	teils-teils	trifft eher nicht zu	trifft nicht zu
Ich bin traurig, dass durch den Tagebau heimische Natur zerstört wird.					
Ich bin besorgt, dass Aspekte dieses Ortes, die ich schätze, verloren gehen (z.B. saubere Luft, schöne Landschaften)					
Ich vermisse das Gefühl von Frieden und Ruhe, das ich früher einmal in dieser Ortschaft genossen habe.					
Ich bin traurig, wenn ich Tagebaulöcher und zerstörte Landschaften betrachte.					
Ich fürchte, dass durch den Tagebau die regionale Landwirtschaft geschädigt wird.					

4. Bitte geben Sie an, ob Sie eine der genannten **Aktivitäten als Reaktion auf die Auswirkungen des Tagebaus** in Ihrer Region durchgeführt haben.

	ja	nein
Meinungsäußerung in Presse oder Rundfunk getätigt		
Meinungsäußerung in sozialen Netzwerken getätigt		
Mit Nachbarschaft, Freundeskreis oder Familie über die Auswirkungen des Tagebaus gesprochen		
Teilnahme an einem Treffen der Dorfgemeinschaft zur Diskussion der Tagebauauswirkungen		
Ich stand aufgrund meiner Bedenken zu den Auswirkungen des Tagebaus in Kontakt mit … politischen Amtsinhaber:innen		
… rechtlichen Vertreter:innen (z.B. Rechtsanwalt) oder Institutionen		
… Umweltbehörden oder -verbänden		
… dem Tagebauunternehmen		
Petition gegen den Ausbau des Tagebaus unterschrieben		
Informationen zu Umwelt- oder gesundheitlichen Auswirkungen eingeholt (z.B. in Internet, Büchern)		
Teilgenommen an lokalen Umwelt- oder Gebäudeschutzmaßnahmen		
Teilgenommen an Demonstrationen gegen den Tagebau		
Unterstützung einer Bürgerinitiative, die sich gegen den Ausbau des Tagebaus einsetzt		

5. Die folgenden Aussagen richten sich nur an Teilnehmende, die eine **Umsiedlung bereits vollständig abgeschlossen** haben.

Alle anderen Teilnehmenden gehen bitte weiter zu Box 6.

Bitte geben Sie an, inwieweit diese Aussagen für Sie zutreffen/nicht zutreffen.

	**trifft zu**	**trifft eher zu**	**teils-teils**	**trifft eher nicht zu**	**trifft nicht zu**
**In meiner neuen Ortschaft fühle ich mich wohler als in meiner alten Ortschaft.**					
**Ich blicke mit einem besseren Gefühl in die Zukunft als vor der Umsiedlung.**					
**Ich hätte mein weiteres Leben gern in meiner alten Ortschaft verbracht.**					
**Ich fühle noch eine tiefe Verbundenheit zu meiner alten Ortschaft.**					
**Ich habe mich innerlich lange gegen die Umsiedlung gewehrt.**					
**Ich fühle mich weiterhin für die Menschen in meiner alten Ortschaft verantwortlich.**					
**Ich wünsche mir, dass meine alte Ortschaft noch für zukünftige Generationen erhalten bleibt.**					
**Ich fühle mich verpflichtet, meine alte Ortschaft noch für zukünftige Generationen zu erhalten.**					
**Durch die Umsiedlungen habe ich Kontakt zu mir liebgewonnenen Menschen verloren.**					
**Die Umsiedlung belastet mich psychisch oder hat mich psychisch belastet.**					
**Ich fühle oder fühlte mich durch die Umsiedlung körperlich erschöpft.**					
**Die Umsiedlung ging für mich mit einer finanziellen Mehrbelastung einher.**					
**Meine berufliche Situation hat sich durch die Umsiedlung verschlechtert.**					
**Die Ausübung meiner Freizeitaktivitäten hat sich durch die Umsiedlung verschlechtert (z.B. Vereinsaktivität, Sport, Spaziergänge).**					
**Durch die Umsiedlung haben sich meine allgemeinen Lebensbedingungen verbessert.**					
**Während des Umsiedlungsprozesses fühlte ich mich von den verantwortlichen Stellen gut informiert und beraten.**					

	trifft zu	trifft eher zu	teils-teils	trifft eher nicht zu	trifft nicht zu
Bitte beantworten Sie die Frage nur, wenn Sie Nutztiere besitzen: **Meine Nutztiere kann ich in der neuen Ortschaft genauso gut halten wie früher.**					
Bitte beantworten Sie die Frage nur, wenn Sie Haustiere besitzen: **Meine Haustiere kann ich in der neuen Ortschaft genauso gut halten wie früher.**					

	ja	nein	keine Angabe
**Die Umsiedlung ging für mich mit dem Verlust von Grundstücksfläche oder Land einher.**			
Bitte beantworten Sie die Frage nur, wenn Sie Nutztiere besitzen: **Vor der Umsiedlung besaßen ich und meine Familie mehr Nutztiere.**			
Bitte beantworten Sie die Frage nur, wenn Sie Haustiere besitzen: **Vor der Umsiedlung besaßen ich und meine Familie mehr Haustiere.**			

6. Die folgenden Aussagen richten sich nur an Teilnehmende, die in einer **Ortschaft mit Umsiedlerstatus** leben, die Umsiedlung also bisher nicht begonnen oder nicht vollständig abgeschlossen haben.

Alle anderen Teilnehmenden gehen bitte weiter zu Box 7.

Bitte geben Sie an, inwieweit diese Aussagen für Sie zutreffen/nicht zutreffen.

	ja	nein	keine Angabe
**Ich weiß bereits, wohin ich umsiedeln werde/würde.**			
**Ich werde voraussichtlich in eine der vorgesehenen Neusiedlungen (z.B. Keyenberg (neu)) umsiedeln.**			
**Das Haus/die Wohnung, in das/die ich umsiedeln werde, ist bereits im Bau oder fertiggestellt.**			
**In meiner neuen Ortschaft bewohne ich vermutlich ein kleineres Grundstück oder verfüge über weniger Land.**			
**Ich werde meine aktuelle Ortschaft voraussichtlich in den nächsten zwei Jahren verlassen.**			

	trifft zu	trifft eher zu	teils-teils	trifft eher nicht zu	trifft nicht zu
**Ich fühle eine tiefe Verbundenheit zu meiner aktuellen Ortschaft.**					
**Ich habe mich innerlich mit dem Gedanken abgefunden, meine Ortschaft zu verlassen.**					
**Ich würde gern in meiner aktuellen Ortschaft alt werden.**					
**Ich empfinde eine gewisse Verantwortung für die Menschen in meiner aktuellen Ortschaft.**					
**Ich wünsche mir, dass meine aktuelle Ortschaft noch für zukünftige Generationen erhalten bleibt.**					
**Ich fühle mich verpflichtet, meine aktuelle Ortschaft noch für zukünftige Generationen zu erhalten.**					
**Ich befürchte, durch die Umsiedlung Kontakt zu mir liebgewonnenen Menschen zu verlieren.**					
**Das Thema Umsiedlung belastet mich psychisch.**					
**Ich fühle mich durch das Thema Umsiedlung körperlich erschöpft.**					
**Ich befürchte, dass eine Umsiedlung für mich mit einer finanziellen Mehrbelastung einhergeht.**					
**Ich befürchte, dass sich meine berufliche Situation durch eine Umsiedlung verschlechtern wird (z.B. längere Arbeitswege).**					
**Ich denke, dass sich durch eine Umsiedlung meine allgemeinen Lebensbedingungen verbessern.**					
**Ich habe das Gefühl, von den verantwortlichen Stellen zur Umsiedlung gut informiert und beraten zu werden.**					
Bitte beantworten Sie die Frage nur, wenn Sie Nutztiere besitzen: **Meine Nutztiere kann ich in der neuen Ortschaft voraussichtlich genauso gut halten wie früher.**					
Bitte beantworten Sie die Frage nur, wenn Sie Haustiere besitzen: **Meine Haustiere kann ich in der neuen Ortschaft voraussichtlich genauso gut halten wie früher.**					

7. Die folgenden Aussagen richten sich nur an Teilnehmende, die in einer **Ortschaft ohne Umsiedlerstatus** am Tagebau leben, also nicht von Umsiedlung betroffen sind.

Alle anderen Teilnehmenden gehen bitte weiter zu Box 8.

Bitte geben Sie an, inwieweit diese Aussagen für Sie zutreffen/nicht zutreffen.

	trifft zu	trifft eher zu	teils-teils	trifft eher nicht zu	trifft nicht zu
**Ich fühle eine tiefe Verbundenheit zu dieser Ortschaft.**					
**Ich empfinde eine gewisse Verantwortung für die Menschen an dieser Ortschaft.**					
**Ich fühle mich verpflichtet, meine Ortschaft noch für zukünftige Generationen zu erhalten.**					
**Die Veränderungen durch den Tagebau beeinflussen meine Lebenssituation negativ.**					
**Aufgrund der Veränderungen durch den Tagebau würde ich meine Ortschaft verlassen, wenn ich könnte.**					

8. Die folgenden Aussagen richten sich an **alle Teilnehmenden**.

Bitte geben Sie an, wie stark Sie sich im Verlauf der letzten **4 Wochen** durch die folgenden **Beschwerden** beeinträchtigt gefühlt haben.

	Nicht beeinträchtigt	Wenig beeinträchtigt	Stark beeinträchtigt
**Bauchschmerzen**			
**Rückenschmerzen**			
**Schmerzen in den Armen, Beinen oder Gelenken (Knie, Hüfte usw.)**			
Nur falls zutreffend: **Menstruationsschmerzen oder andere Probleme bei der Menstruation**			
**Schmerzen oder Probleme beim Geschlechtsverkehr**			
**Kopfschmerzen**			
**Schmerzen im Brustbereich**			
**Schwindel**			
**Ohnmachtsanfälle**			
**Herzklopfen oder Herzrasen**			
**Kurzatmigkeit**			
**Verstopfung, nervöser Darm oder Durchfall**			

	Nicht beeinträchtigt	Wenig beeinträchtigt	Stark beeinträchtigt
**Übelkeit, Blähungen oder Verdauungsbeschwerden**			
**Schwierigkeiten, ein- oder durchzuschlafen, oder vermehrter Schlaf**			
**Müdigkeit oder das Gefühl, keine Energie mehr zu haben**			

9. Die folgenden Aussagen richten sich an **alle Teilnehmenden**.
Bitte geben Sie an, wie oft Sie sich im Verlauf der letzten **2 Wochen** durch die folgenden **Beschwerden** beeinträchtigt gefühlt haben.

	Überhaupt nicht	An einzelnen Tagen	An mehr als der Hälfte der Tage	Beinahe jeden Tag
**Nervosität, Ängstlichkeit oder Anspannung**				
**Nicht in der Lage sein, Sorgen zu stoppen oder zu kontrollieren**				
**Übermäßige Sorgen bezüglich verschiedener Angelegenheiten**				
**Schwierigkeiten zu entspannen**				
**Rastlosigkeit, so dass Stillsitzen schwer fällt**				
**Schnelle Verärgerung oder Gereiztheit**				
**Gefühl der Angst, so als würde etwas Schlimmes passieren**				
**Wenig Interesse oder Freude an Ihren Tätigkeiten**				
**Niedergeschlagenheit, Schwermut oder Hoffnungslosigkeit**				
**Schwierigkeiten, ein- oder durchzuschlafen, oder vermehrter Schlaf**				
**Müdigkeit oder das Gefühl, keine Energie mehr zu haben**				
**Verminderter Appetit oder das übermäßige Bedürfnis zu essen**				
**Schlechte Meinung von sich selbst; Gefühl ein Versager zu sein oder die Familie enttäuscht zu haben**				
**Schwierigkeiten, sich auf etwas zu konzentrieren, z.B. beim Zeitungslesen oder Fernsehen**				
**Waren Ihre Bewegung und Ihre Sprache so verlangsamt, dass es auch anderen auffallen würde? Oder waren Sie im Gegenteil “zappelig” oder ruhelos und hatten dadurch einen stärkeren Bewegungsdrang als sonst?**				
**Gedanken, dass Sie lieber tot wären oder sich Leid zufügen möchten**				

10. Bitte nutzen Sie für weiterführende **Anmerkungen, Erklärungen oder Kommentare** zu Ihrer persönlichen Situation oder zum Fragebogen das folgende Freitextfeld (optional):

Sollten Sie beim Ausfüllen dieses Fragebogens bemerkt haben, dass Sie sich durch die Auswirkungen des Tagebaus und Veränderungen Ihrer Heimat gesundheitlich stark belastet fühlen und möglicherweise professionelle Unterstützung benötigen, wenden Sie sich bitte an Dr. Andrea Kaifie-Pechmann (Institut für Arbeits-, Sozial und Umweltmedizin, Uniklinik RWTH Aachen) zur weiteren Beratung: [redacted] oder [redacted].

Vielen Dank für Ihre Teilnahme an dieser Studie!

Bitte senden Sie den Fragebogen bis spätestens 23.07.2021 in dem vorfrankierten Umschlag zurück an:

PD Dr. med. Andrea Kaifie-Pechmann und Theresa Krüger

Institut für Arbeits-, Sozial- und Umweltmedizin

Uniklinikum RWTH Aachen

Pauwelsstraße 30

52074 Aachen
